# Integrated ATAC-seq and mRNA-seq analyses on granulosa cells identify key regulators of follicle selection in chickens

**DOI:** 10.1186/s40104-026-01386-y

**Published:** 2026-04-17

**Authors:** Dandan Li, Chao Qi, Yi Sun, Li Kang, Qingqing Wei, Yunliang Jiang

**Affiliations:** 1 College of Animal Science and Technology, Shandong Agricultural University, 7 Panhe Street, Taian, 271017 China; 2 Shandong Provincial Animal Husbandry Station, Jinan, 250010 China; 3 Shandong Provincial Key Laboratory for Livestock Germplasm Innovation & Utilization, Shandong Agricultural University, Taian, 271017 China

**Keywords:** ATAC-seq, Chicken, Follicle selection, Granulosa cell, mRNA-seq

## Abstract

**Background:**

The differences in egg production performance among hens are closely linked to the efficiency of follicle selection, which is characterized by granulosa cell differentiation and progesterone production. In this study, by integrating ATAC-seq and mRNA-seq analyses on granulosa cells from pre-hierarchical (Pre-GCs) and hierarchical (Post-GCs) follicles, we set out to identify key regulatory factors involved in chicken follicle selection.

**Results:**

ATAC-seq analysis revealed 21,616 upregulated and 6,344 downregulated differentially accessible peaks in Post-GCs compared to Pre-GCs. Enrichment analysis of the top 400 upregulated and downregulated peaks (ranked by |log_2_FC|) identified 106 upregulated and 81 downregulated genes encoding transcription factors (TFs). mRNA-seq analysis showed 1,337 upregulated and 2,282 downregulated differentially expressed genes in Post-GCs compared to Pre-GCs. Among these, 146 genes were uniquely expressed in Pre-GCs, and 40 genes were uniquely expressed in Post-GCs. Integrated ATAC-seq and mRNA-seq analyses revealed that chromatin accessibility and gene expression were not always positively correlated. Additionally, four significantly upregulated core TFs (*CREM*, *ESRRB*, *NR5A1*, *GATA6*) and one significantly downregulated TF (*ETS1*) genes were identified. The upregulated TFs were associated with 651 significantly upregulated target genes across both mRNA and ATAC-seq data, while the downregulated TFs were associated with 236 significantly downregulated target genes. KEGG enrichment of these target genes identified core genes enriched in pathways closely related to follicle selection, such as Wnt signaling pathway. In the protein–protein interaction (PPI) network of core upregulated target genes, genes such as *SMAD2* and *PPARA* occupied central positions, while genes such as *LEF1* and *RAC2* were central in the PPI network of core downregulated target genes. Among these core TFs, *GATA6* is confirmed to promote the expression of key cholesterol and progesterone synthesis genes to enhance both cholesterol and progesterone production, with follicle-stimulating hormone further amplifying its effect on progesterone synthesis.

**Conclusions:**

Our integrated ATAC-seq and mRNA-seq analyses define key epigenetic and transcriptional changes during chicken follicle selection. We highlight core transcription factors, including CREM, ESRRB, NR5A1, GATA6 and ETS1, and their stage-specific target networks. Functional assays show that GATA6 promotes cholesterol and progesterone synthesis, providing mechanistic insight and candidate targets for improving hen reproductive performance.

**Supplementary Information:**

The online version contains supplementary material available at 10.1186/s40104-026-01386-y.

## Introduction

Poultry is a major sector of China's livestock industry, and improving laying performance is critical for both economic growth and food security. In hens, egg production is governed by the hypothalamic–pituitary–gonadal axis, with follicular development and selection serving as direct determinants [1]. Chicken follicle development involves two key recruitment steps: formation of primordial follicles and the cyclic selection of one small yellow follicle (SYF) into the hierarchical follicles (also known as the preovulatory follicles, F1–F6), which then rapidly grow to ovulation [2]. Although sexually mature hens possess many oocytes, only a minority are selected and ovulated; thus, follicle selection efficiency closely correlates with variation in laying performance [3].

Granulosa cells play a pivotal role in chicken follicle selection. Before follicle selection, granulosa cells are undifferentiated and exhibit limited steroidogenic capacity [4]. Following follicle selection, granulosa cells differentiate to form a tightly packed monolayer cubic structure, shift from follicle stimulating hormone (FSH) dependence to luteinizing hormone (LH) responsiveness and secrete large amounts of progesterone by upregulating steroidogenic genes and various paracrine factors [5–7]. These shifts occur alongside multifaceted molecular changes, notably in epigenetics, transcription, and metabolism [8]. Granulosa cells of chicken ovarian follicles also provide nutrients and signals to the oocyte through direct exchange of materials; their proliferation, differentiation, and apoptosis directly determine follicular fate, which has also been documented in mammals [9–11]. However, the functional transition of granulosa cells during follicle selection and its underlying molecular mechanisms remain poorly understood, particularly in terms of differences in chromatin accessibility and transcriptomic regulation. How these changes influence gene expression, cellular function, and follicular development is not fully elucidated.

Advances in high-throughput sequencing have enabled multi-omics dissection of follicle selection, with approaches such as combined transcriptomics and proteomics [12], as well as transcriptomics and metabolomics [13], revealing candidate regulators and pathways. Several foundational transcriptomic studies identified genes and signaling pathways associated with follicle development or regression [12, 14, 15], and recent single-cell transcriptome analysis resolved the dynamics of granulosa cells across selection and atresia [16]. However, systematic evidence at the epigenetic level, particularly how changes in enhancer/promoter accessibility and upstream transcription factors (TFs) activity shape these regulatory programs, remains scarce in poultry, especially in the context of follicle selection. To address this gap, we performed integrative ATAC-seq and mRNA-seq analyses on granulosa cells of pre-hierarchical (Pre-GCs) and hierarchical (Post-GCs) follicles, thereby offering a comprehensive view of the regulatory differences between Pre-GCs and Post-GCs at both the epigenetic and transcriptomic levels, which may contribute to understanding their functional significance in follicle selection.

## Materials and methods

### Animals and sample collection

Hy-Line Brown laying hens aged 30 weeks with consistent laying records were randomly selected from the breeding facility affiliated with Shandong Agricultural University. Hens were maintained under uniform husbandry conditions, provided with unrestricted access to feed and water, and subjected to a 16 h light and 8 h dark photoperiod. Chickens were euthanized via cervical dislocation, after which small white follicles (SWFs), large white follicles (LWFs), SYFs, and F6 to F1 were rapidly harvested from the ovaries and placed in cold phosphate-buffered saline. All procedures involving animals were reviewed and approved by the Institutional Animal Care and Use Committee of Shandong Agricultural University (Approval No. SDAUA-2024-088) and were performed in accordance with the Ministry of Science and Technology of China's "Guidelines for Experimental Animals".

### Cell culture and treatment

Cells were prepared and cultured according to the method of Hu et al. [17]. In brief, Pre-GCs (from SWFs, LWFs, and SYFs) were digested with 1 mg/mL collagenase II (Coolaber, Beijing, China) in a 37 °C water bath for 5 min, while Post-GCs (including F6–F1) were digested with 0.25% trypsin-EDTA (Gibco, Grand Island, NY, USA) in a 37 °C water bath for 8 min, after which both cell suspensions were filtered through a 200-mesh sieve. Cells were then harvested by centrifugation at 556 × *g* for 5 min and resuspended in M199 medium (Gibco, Grand Island, NY, USA) supplemented with fetal bovine serum (FBS; Vazyme, Nanjing, China) and 1% antibiotic solution (Solarbio, Beijing, China). Pre-GCs were cultured in 1% FBS, whereas Post-GCs were grown in 5% FBS. All cells were seeded at 1 × 10^6^ cells per well in six-well plates and incubated at 39 °C with 5% CO_2_.

### Plasmid construction and cell treatment

The full-length coding sequence of chicken *GATA6* was inserted into the *Nhe* I and *Xho* I restriction sites of the pcDNA3.1(+) vector, and the construct was verified by DNA sequencing. For FSH treatment, when cells reached 90% confluence, the medium was replaced with fresh culture medium containing different concentrations of FSH (MedChemExpress, Shanghai, China), and cells were incubated for 24 h. For the combined treatment, when cells reached 80% confluence, plasmid transfection was performed using Lipofectamine LTX and Plus Reagent (Thermo Fisher Scientific, Waltham, MA, USA); after 6 h, the medium was replaced with fresh culture medium with or without FSH (50 ng/mL) and further incubated for 24 h.

### ATAC library preparation and sequencing

For ATAC-seq, three biological replicates were prepared for each group, including Pre-GCs (samples Pre-GCs-A1 to A3) and Post-GCs (samples Post-GCs-A1 to A3). The ATAC-seq library was prepared in the following steps: First, the cell samples were thawed and centrifuged. Then, a lysis buffer was added to resuspend the cells, and nuclei were collected by centrifugation. Next, Tn5 transposase reaction was performed using the TruePrep^®^ DNA Library Prep Kit (Vazyme, Nanjing, China). The reaction was carried out at 37 °C for 30 min, followed by purification of the product using DNA beads. Subsequently, PCR enrichment was performed, again using the TruePrep^®^ DNA Library Prep Kit (Vazyme, Nanjing, China) to prepare the reaction system. After amplification, the library was purified again using magnetic beads. The library quality was assessed using Agilent 2100 Bioanalyzer (Agilent, Santa Clara, CA, USA), and once it passed quality control, sequencing was performed on the Illumina sequencing platform by Genedenovo Biotechnology Co., Ltd. (Guangzhou, China).

### ATAC-seq data processing

After sequencing, the following analyses were performed: 1) Quality control of raw data was conducted using fastp (Version: 0.19.5) [18] to filter low-quality reads; 2) Clean reads were aligned to the reference genome GRCg7b (https://www.ncbi.nlm.nih.gov/datasets/genome/GCF_016699485.2) using Bowtie2 (Version: 2.2.8) [19]; 3) Peak calling was performed using MACS2 (Version: 2.1.2) [20] with a *q*-value threshold of < 0.05; 4) DiffBind (Version: 2.8) [21] was used to merge peaks between groups and perform differential peak analysis (|log_2_FC| > 1, FDR < 0.05); 5) Gene Ontology (GO) (http://www.geneontology.org/) and Kyoto Encyclopedia of Genes and Genomes (KEGG) (https://www.genome.jp/kegg/) functional enrichment analyses were performed on differential peaks corresponding genes; 6) Motif prediction for TFs was performed using the AME (Analysis of Motif Enrichment) tool from the MEME Suite (http://meme-suite.org/).

### RNA isolation, library preparation, and sequencing

For mRNA-seq, three biological replicates were used for each group, comprising Pre-GCs (samples Pre-GCs-R1 to R3) and Post-GCs (samples Post-GCs-R1 to R3). Total RNA was extracted from cells using the TRIzol method (Accurate Biotechnology, Hunan, China). RNA quality was strictly assessed by agarose gel electrophoresis, NanoDrop (Thermo Fisher Scientific, Waltham, MA, USA), and Agilent 2100 Bioanalyzer (Agilent, Santa Clara, CA, USA). mRNA libraries were constructed using the Hieff NGS^®^ Ultima Dual-mode mRNA Library Prep Kit (Yeasen, Shanghai, China). After the sequencing libraries passed quality control, sequencing was performed on the Illumina sequencing platform by Genedenovo Biotechnology Co., Ltd. (Guangzhou, China).

### mRNA-seq data analysis

The specific analysis process is as follows: 1) Quality control of raw data was performed using fastp (Version: 0.18.0) [18] to filter low-quality reads; 2) Clean reads were aligned to the reference genome GRCg7b (https://www.ncbi.nlm.nih.gov/datasets/genome/GCF_016699485.2/) using HISAT2 (Version: 2.1.0) [22]; 3) Transcripts were reconstructed using StringTie (Version: 1.3.4) [23] and gene expression levels (TPM) were calculated with RSEM (Version: 1.2.19) [24]; 4) Differential expression analysis was conducted using DESeq2 (Version: 1.20.0) [25] (|log_2_FC| > 1, FDR < 0.05); 5) GO (http://www.geneontology.org/) and KEGG (http://www.kegg.jp) functional enrichment analyses were performed on the differentially expressed genes (DEGs).

### Integration of ATAC-seq and mRNA-seq

To systematically investigate the concordant regulatory patterns between ATAC-seq and mRNA-seq data, we integrated chromatin accessibility profiles with gene expression profiles by mapping ATAC-seq peak regions to their corresponding genes. For intergroup differential analysis, chromatin accessibility peaks were categorized as proximal (gene body plus 0–2 kb upstream) or distal (2–100 kb upstream), and annotated with corresponding genes. TFs enriched in ATAC-seq differential peaks were intersected with DEGs identified in the mRNA-seq dataset to identify core TFs. To determine key target genes, we intersected the predicted targets of key TFs with genes that were significantly upregulated or downregulated in both datasets. KEGG pathway enrichment analysis was conducted on selected key target genes to explore their involvement in relevant biological processes and pathways. Finally, protein–protein interaction (PPI) networks were constructed using the STRING database [26] (http://string-db.org).

### RNA extraction and reverse transcription real-time quantitative PCR (RT-qPCR)

Total RNA was extracted using the RNA Simple Total RNA Kit (TIANGEN, Beijing, China). RNA was DNase-treated and reverse-transcribed using Evo M-MLV RT Mix with gDNA Clean (Accurate Biotechnology, Hunan, China) following the manufacturer's instructions. qPCR was performed with SYBR Green Premix Pro Taq HS qPCR Kit (Accurate Biotechnology, Hunan, China) on an Archimed X4 thermocycler (ROCGENE, Beijing, China). Relative expression was calculated by the 2^−ΔΔCt^ method [27] with *GAPDH* as the internal control. Primer sequences are listed in Table S1.

### Enzyme-linked immunosorbent assay (ELISA)

The culture supernatant of Pre-GCs was collected 24 h after transfection or FSH treatment. Progesterone levels were quantified using a commercial chicken progesterone ELISA kit (MEIMIAN, Yancheng, China) following the manufacturer's protocol. Meanwhile, cholesterol levels were measured using the chicken total cholesterol ELISA kit (Enzyme-linked Biotechnology, Shanghai, China).

### Statistical analysis

Each experiment consisted of at least three biological replicates. Results are presented as the mean ± standard error of the mean (SEM). For two-group comparisons, Student’s* t*-test was used. For comparisons involving more than two groups, one-way ANOVA followed by LSD's multiple comparison was applied using SPSS 25.0 (SPSS Inc., Chicago, IL, USA), with statistical significance set at* P* < 0.05. Graphs were generated using GraphPad Prism software (version 8.0; San Diego, CA, USA).

## Results

### ATAC-seq data quality assessment and chromatin accessibility overview

We first assessed the alignment statistics of each sample to the reference genome (Table S2) and calculated the fraction of reads in peaks (FRiP) values (Table S3). All samples showed high unique mapping rates, low duplication rates, and FRiP values ranging from 32.29% (Pre-GCs-A2) to 43.93% (Post-GCs-A3). These results indicate that the sequencing data are of high quality and suitable for downstream chromatin accessibility analysis. Further analysis revealed that the ATAC-seq libraries displayed typical fragment size distributions: a prominent nucleosome-free peak at approximately 50 bp, and periodic mono-, di-, and tri-nucleosome peaks around 200, 400, and 600 bp, respectively, confirming successful library construction (Fig. S1A). Read density analysis around ± 2 kb of transcription start sites (TSS) showed sharp peaks, suggesting accurate signal localization (Fig. S1B). Genomic annotation of peaks revealed that most of them were located in intronic and distal intergenic regions, with a smaller proportion in promoter regions; the overall composition was similar between the two groups (Fig. S1C). Principal component analysis (PCA) and Pearson correlation analyses demonstrated strong within-group reproducibility and clear separation between Pre-GCs-A and Post-GCs-A (Fig. S1D and E).

### ATAC-seq reveals dynamic chromatin accessibility during follicle selection

To explore the regulatory basis of follicle selection, we identified a total of 91,459 differential peaks between Pre-GCs-A and Post-GCs-A. Among them, 27,960 peaks were found to be significantly different (|log_2_FC| > 1, FDR < 0.05), including 21,616 upregulated and 6,344 downregulated peaks in Post-GCs-A (Fig. S2A and B). The Venn diagram showed that 5,680 DAPs were shared between the two groups, 5,250 were specific to Pre-GCs-A, and 17,030 were specific to Post-GCs-A, suggesting a marked expansion of open chromatin regions as follicle selection progresses (Fig. S2C).

### Functional enrichment analysis of genes associated with DAPs

**Fig. 1:**
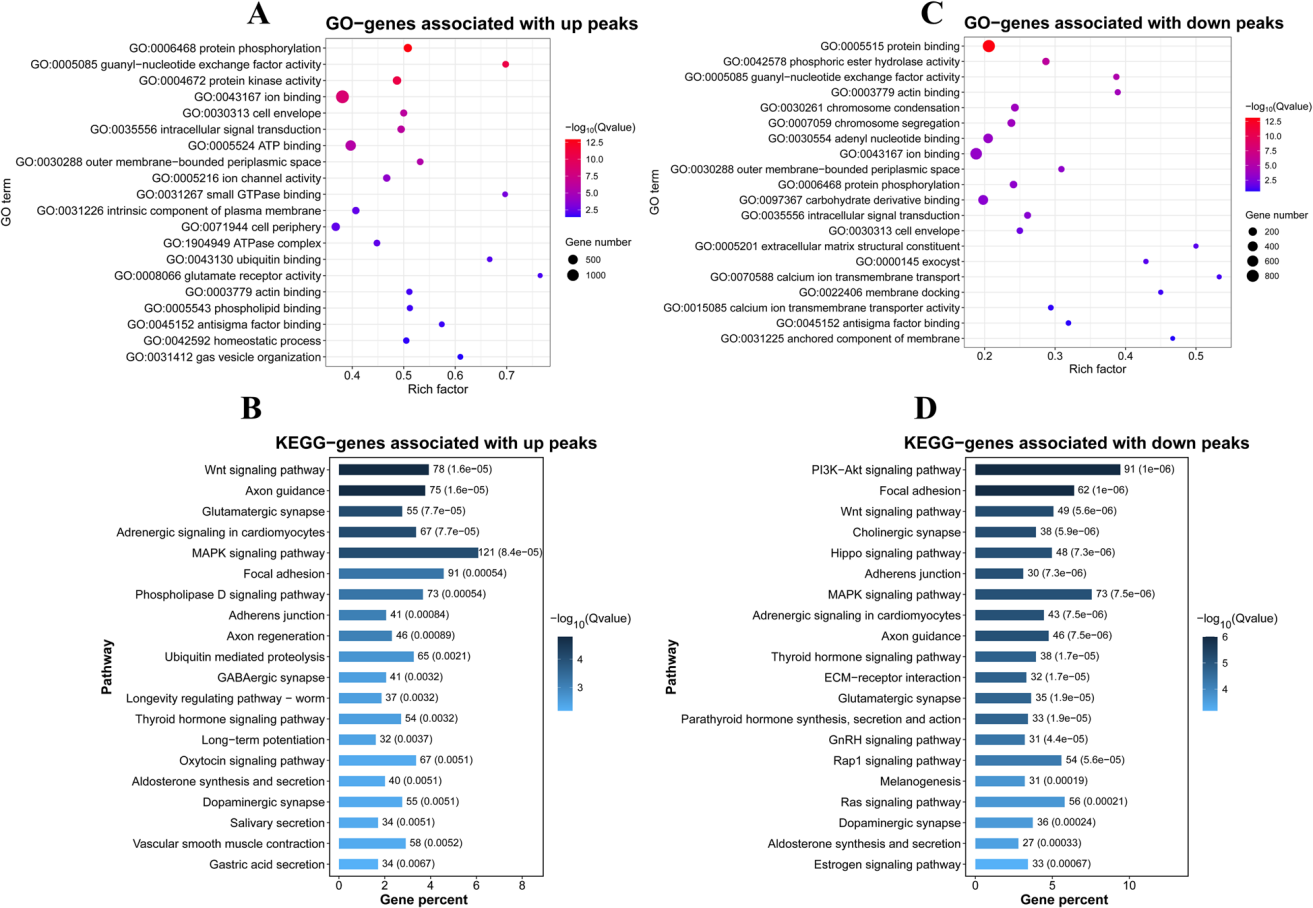
Functional enrichment analysis of ATAC-seq peak-associated genes. **A** and **B** GO terms and KEGG pathways enrichment analyses of genes associated with upregulated DAPs. **C** and **D** GO terms and KEGG pathways enrichment analyses of genes associated with downregulated DAPs

We performed GO and KEGG enrichment analyses on genes associated with the DAPs. For the upregulated DAPs, GO analysis revealed significant enrichment in terms related to protein phosphorylation, protein kinase activity, intracellular signal transduction, among others (Fig. 1A). KEGG analysis revealed enrichment in key biological pathways, including Wnt signaling pathway, MAPK signaling pathway, and adherens junction (Fig. 1B). In contrast, genes associated with downregulated accessible regions were enriched in GO terms such as adenyl nucleotide binding, protein binding, and chromosome segregation (Fig. 1C). KEGG analysis revealed enrichment in several signaling pathways, including PI3K-Akt signaling, GnRH signaling, and other pathways related to intracellular signaling regulation (Fig. 1D).

### Analysis of transcription factor motifs in DAPs identified by ATAC-seq

**Fig. 2:**
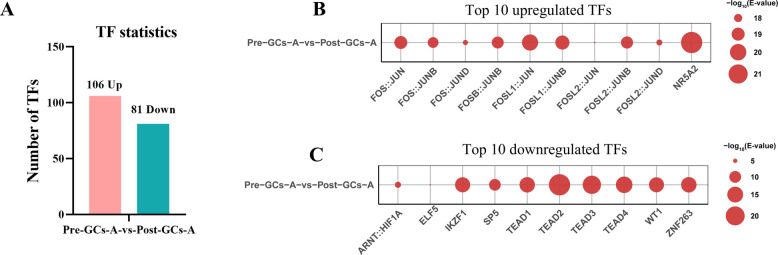
Motif enrichment analysis of TFs in DAPs identified by ATAC-seq. **A** Number of enriched TFs in upregulated and downregulated DAPs. Top 10 TFs enriched in upregulated (**B**) or downregulated (**C**) DAPs

To explore the regulatory mechanisms of chromatin accessibility changes between Pre-GCs-A and Post-GCs-A, we performed motif enrichment analysis on the top 400 upregulated and 400 downregulated DAPs (ranked by |log_2_FC|) using the AME tool from the MEME Suite. Motif analysis revealed enrichment of 106 TFs in upregulated DAPs and 81 TFs in downregulated DAPs (Fig. 2A). Among the upregulated TFs, several members of the activator protein 1 (AP-1) family (e.g., FOS::JUN, FOSL1::JUN, and FOSL2::JUNB) exhibited high activity (Fig. 2B). The nuclear receptor subfamily 5 group A member 2 (NR5A2) showed the highest enrichment (Fig. 2B). Conversely, the downregulated TFs include transcriptional enhancer factor domain family members 1–4 (TEAD1–4), Ikaros family zinc finger 1 (IKZF1), Wilms tumor 1 (WT1), among others (Fig. 2C).

### Overview of mRNA-seq data quality and differential gene expression

To integrate chromatin accessibility with gene expression changes, we conducted mRNA-seq analysis on the same Pre-GCs and Post-GCs samples that underwent ATAC-seq, aiming to explore gene expression changes in Pre-GCs and Post-GCs. The mRNA-seq data showed high quality, with over 97% of reads mapped to the reference genome (Table S4). Sequencing depth for each sample ranged from 5.13 × to 7.74 ×, which meets the data requirement for mRNA-seq analysis (Table S5). The sequencing depth and base composition statistics further confirm the robustness of the sequencing data. Additionally, Table S6 summarizes the number of reference genes, novel genes, and total genes detected in each sample. The gene detection rates for each sample ranged from 81.01% to 83.65%, reflecting high sequencing efficiency and successful data acquisition across all samples (Table S6). PCA and correlation analyses revealed clear separation between Pre-GCs-R and Post-GCs-R, along with tight clustering and high within-group consistency (Fig. S3A and B). Differential expression analysis identified 1,337 upregulated and 2,282 downregulated genes (FDR < 0.05, |log_2_FC| > 1) (Fig. S3C), with their distribution visualized by a volcano plot (Fig. S3D).

### Functional annotation and expression profiling of DEGs identified by mRNA-seq

**Fig. 3:**
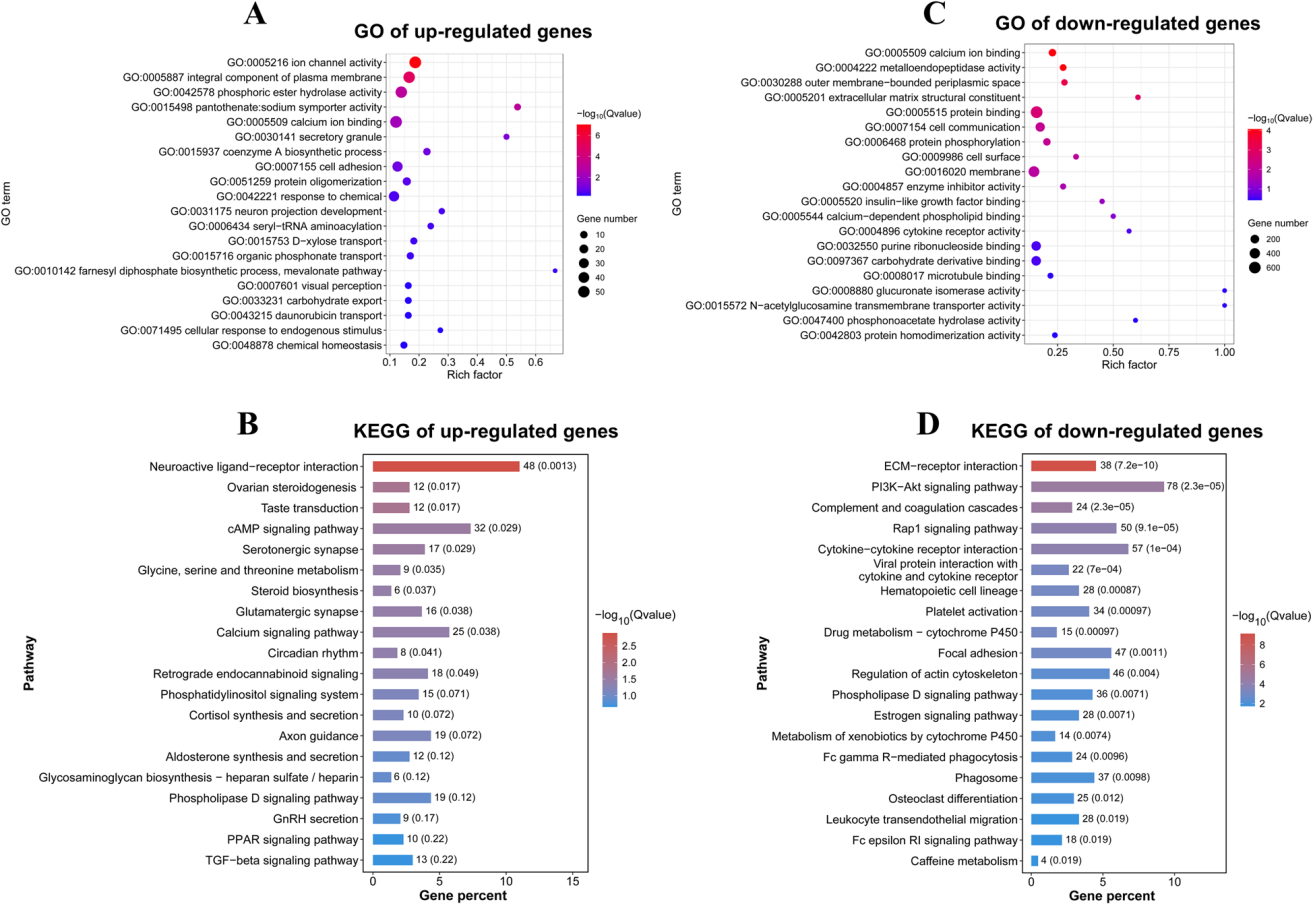
Functional enrichment analysis of DEGs from mRNA-seq. **A** and **B** GO term and KEGG pathway enrichment analyses of upregulated DEGs. **C** and **D** GO term and KEGG pathway enrichment analyses of downregulated DEGs

To elucidate the biological roles of the DEGs, we performed GO and KEGG enrichment analyses. The upregulated genes in the Post-GCs-R group were significantly enriched in GO terms such as cell adhesion and calcium ion binding (Fig. 3A). KEGG pathway analysis revealed their involvement in neurotransmitter-related pathways (e.g., serotonergic synapse and glutamatergic synapse), ovarian steroidogenesis, cAMP signaling pathway, among others (Fig. 3B). Conversely, downregulated genes were primarily associated with metallopeptidase activity, outer membrane-bounded periplasmic space, and cell communication in the GO analysis (Fig. 3C), while KEGG pathways analysis indicated significant enrichment in PI3K-Akt signaling, estrogen signaling pathway, among others (Fig. 3D).

**Fig. 4:**
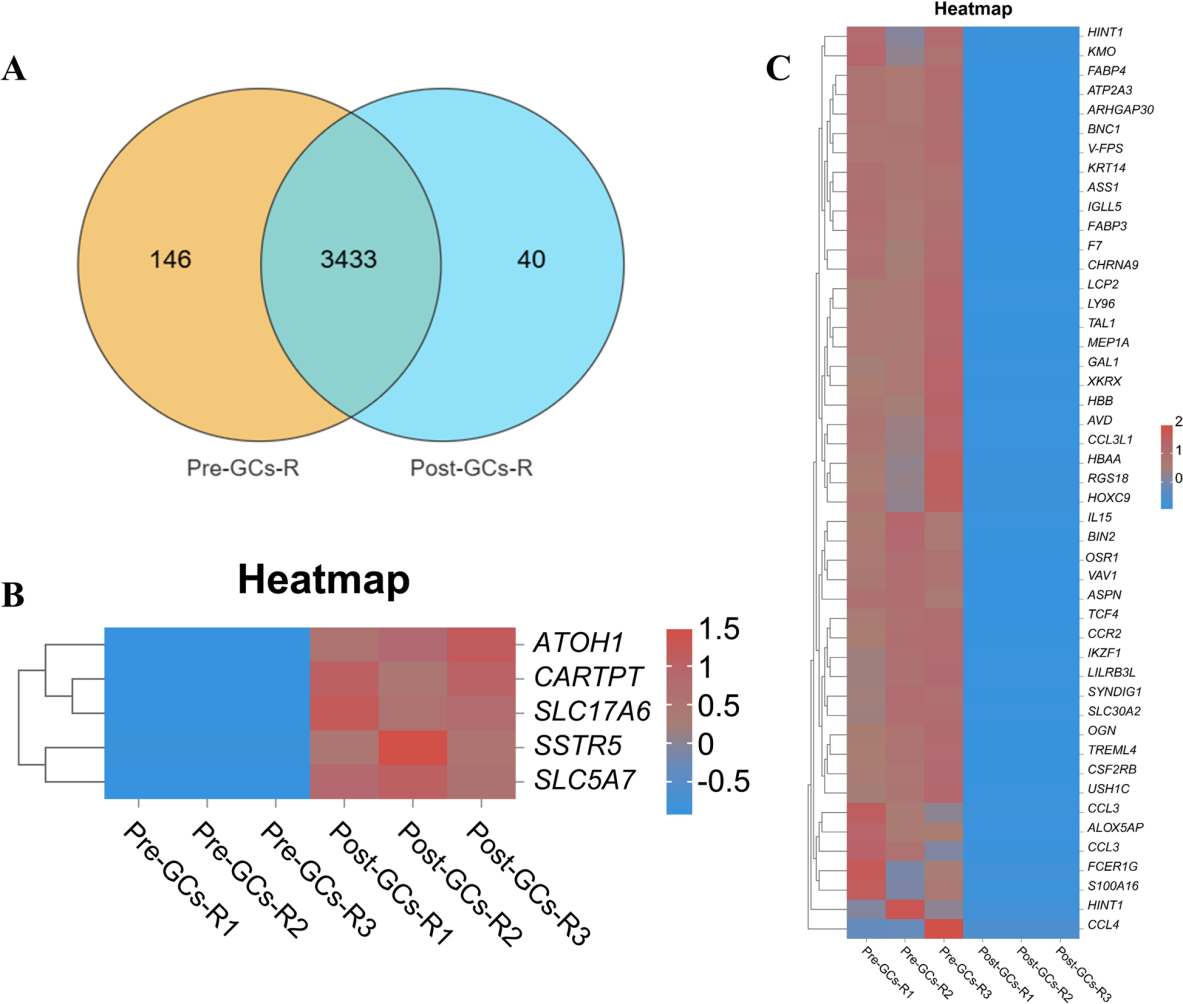
Expression pattern analysis of DEGs identified by mRNA-seq. **A** Venn diagram showing the number of shared and unique DEGs between Pre-GCs-R and Post-GCs-R. **B** and **C** Heatmaps of representative DEGs specifically expressed in Post-GCs-R (**B**) and Pre-GCs-R (**C**)

Further Venn diagram analysis identified 3,433 shared DEGs between Pre-GCs-R and Post-GCs-R, along with 146 DEGs (Table S7) specific to Pre-GCs-R and 40 DEGs (Table S8) specific to Post-GCs-R (Fig. 4A). Heatmap analysis further revealed distinct expression patterns of several key genes between the two stages (Fig. 4B and C), such as cocaine- and amphetamine-regulated transcript prepropeptide (*CARTPT*), somatostatin receptor 5 (*SSTR5*), interleukin 15 (*IL15*), and C–C motif chemokine receptor 2 (*CCR2*), suggesting their potential roles in the functional transition of granulosa cells during follicle selection.

### Integrative analysis of ATAC-seq and mRNA-seq reveals coordinated changes in chromatin accessibility and gene expression

**Fig. 5:**
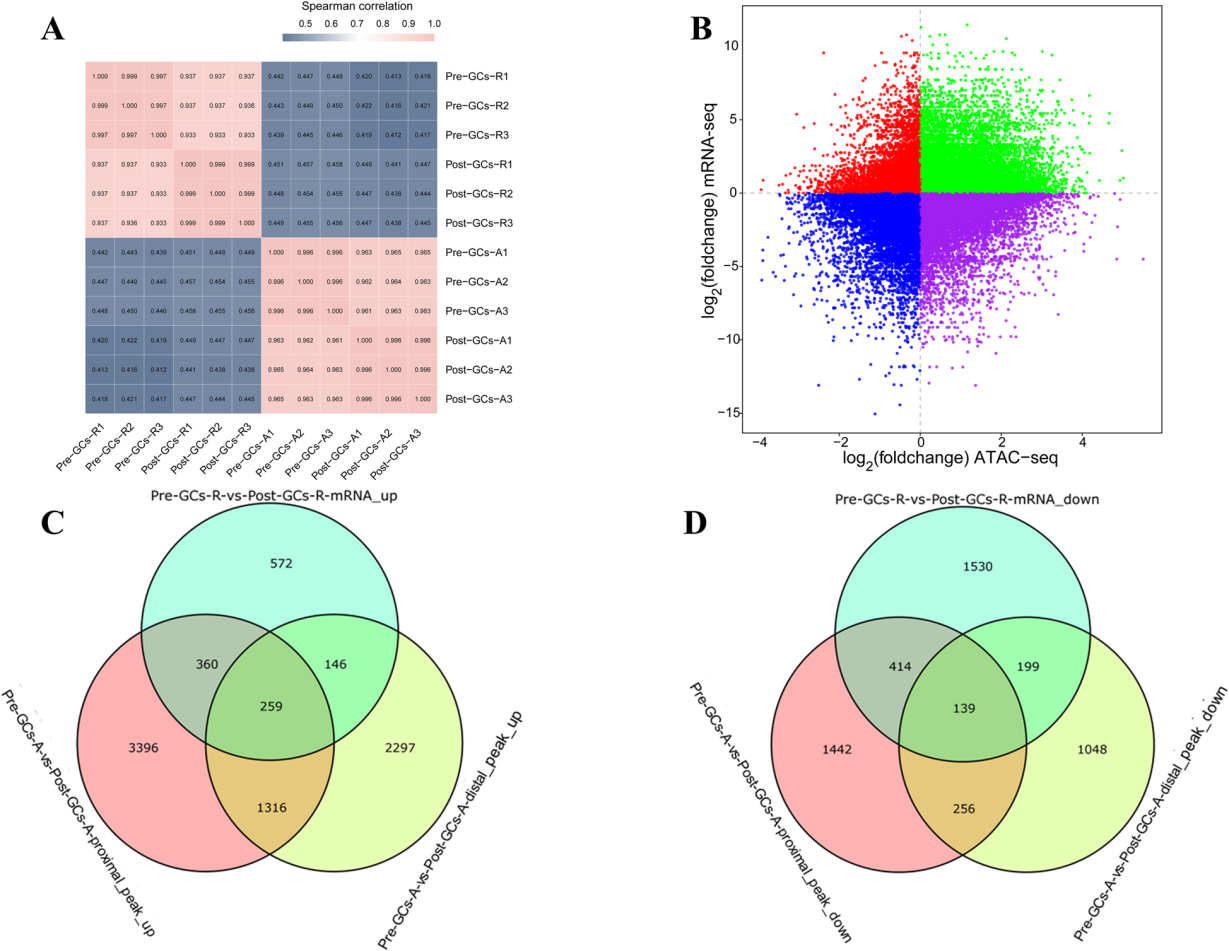
Integration of ATAC-seq and mRNA-seq data reveals changes in chromatin accessibility and gene expression. **A** Overall correlation analysis between chromatin accessibility and transcriptional activity across samples. **B** Four-quadrant plot showing the relationship between changes in chromatin accessibility and gene expression. Each dot represents a gene and its associated chromatin region, with colors indicating different trends in expression and accessibility. **C** Venn diagram showing the overlap between upregulated genes (mRNA-seq) and genes associated with increased chromatin accessibility in proximal and distal regions. **D** Venn diagram showing the overlap between downregulated genes (mRNA-seq) and genes associated with decreased chromatin accessibility in proximal and distal regions

To explore the relationship between chromatin accessibility and transcriptional activity, we performed a joint analysis of ATAC-seq and mRNA-seq data from Pre-GCs and Post-GCs samples. As shown in Fig. 5A, Spearman correlation heatmaps revealed high consistency between ATAC-seq and mRNA-seq data within each group, while correlations between groups were relatively low. These results indicate good reproducibility and support the reliability of cross-omics integration. In the four-quadrant plot (Fig. 5B), each point represents a gene and its corresponding chromatin region. The green (Table S9) and blue (Table S10) points represent cases where chromatin accessibility and gene expression are both either upregulated or downregulated. In contrast, the red and purple points reflect discordant trends. These results suggest that while many genes exhibit coordinated changes in chromatin accessibility and expression, others show discordant patterns, implying that additional layers of regulation may influence gene expression beyond chromatin accessibility. To further investigate the regulatory effects of chromatin accessibility on gene expression, we analyzed the overlap between DEGs and genes associated with DAPs in proximal and distal regions. Figure 5C shows that 259 transcriptionally upregulated genes were linked to increased chromatin accessibility in both proximal and distal regions, suggesting coordinated regulation across multiple regulatory elements. Conversely, 139 downregulated genes were associated with decreased chromatin accessibility in both regions (Fig. 5D), indicating that reduced expression may result from declining accessibility at multiple regulatory sites. These overlaps support the notion that transcriptional changes are partially driven by chromatin accessibility dynamics.

### Identification of key TFs and their putative target genes via multi-omics analysis

**Fig. 6:**
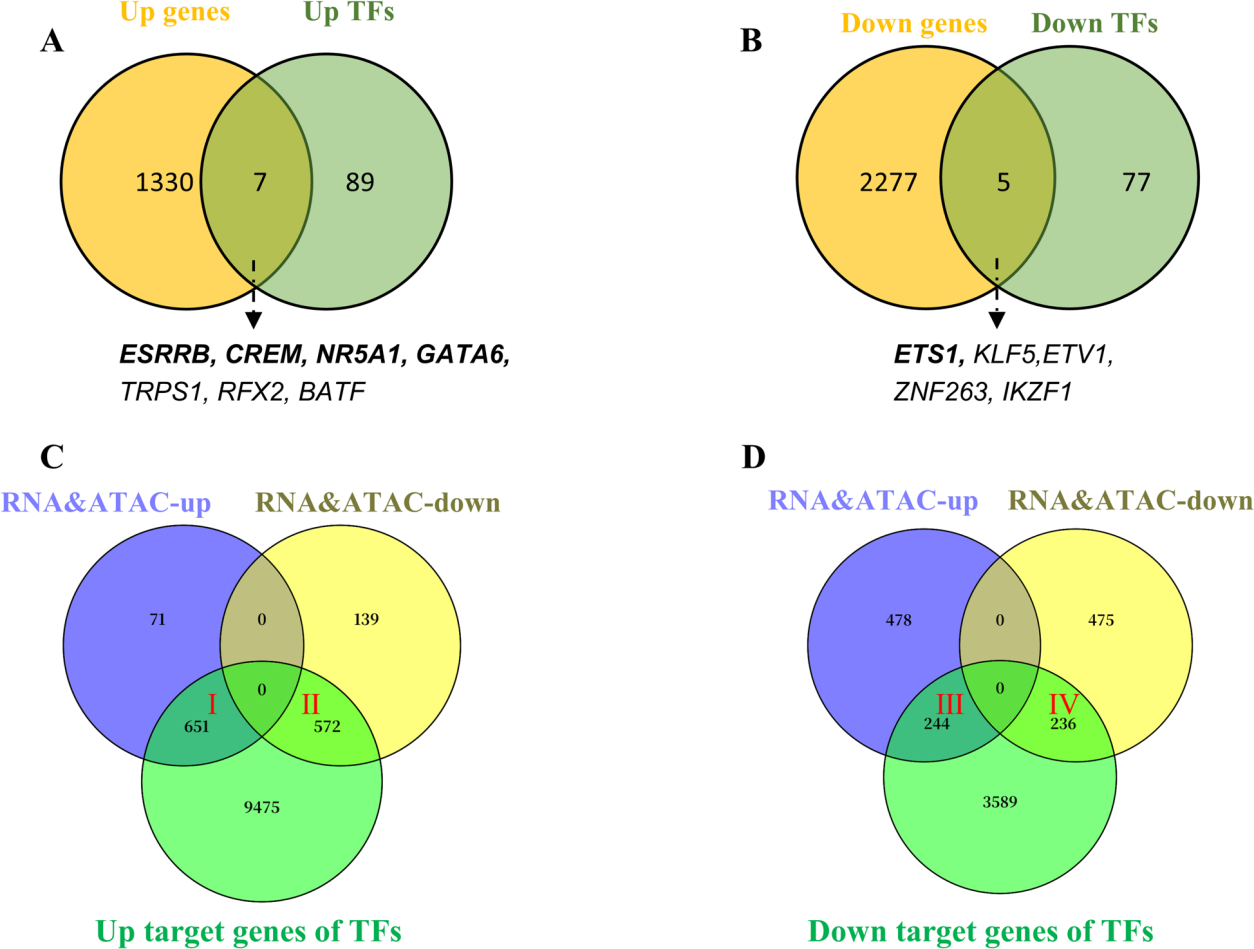
Integrative identification of key TFs and their target genes based on ATAC-seq and mRNA-seq data. **A** Venn diagram showing the overlap between significantly upregulated genes and TFs enriched in upregulated ATAC-seq peaks. **B** Venn diagram showing the overlap between significantly downregulated genes and TFs enriched in downregulated ATAC-seq peaks. **C** and **D** Venn diagrams showing the intersection between predicted target genes of upregulated (**C**) or downregulated (**D**) TFs and genes jointly up- or downregulated in both mRNA-seq and ATAC-seq datasets. Up/Down Genes refer to genes significantly up- or downregulated in mRNA-seq. Up/Down TFs are TFs enriched in ATAC-seq peaks with increased or decreased chromatin accessibility. RNA&ATAC-Up and RNA&ATAC-Down indicate genes simultaneously up- or downregulated in both mRNA-seq and ATAC-seq datasets

To identify core TFs with potential regulatory roles, we integrated ATAC-seq motif enrichment results (Fig. 2) with mRNA-seq differential expression data (Fig. S3C). Seven TFs were both enriched in upregulated ATAC-seq peaks and significantly upregulated at the transcriptional level (Fig. 6A), suggesting potential roles in transcriptional activation. Conversely, five TFs were enriched in downregulated peaks and downregulated in mRNA-seq (Fig. 6B), implying possible involvement in transcriptional repression or inhibition pathways. Based on literature evidence, we prioritized four upregulated TFs (estrogen-related receptor beta (ESRRB), nuclear receptor subfamily 5 group A member 1 (NR5A1), GATA6, and cyclic adenosine monophosphate responsive element modulator (CREM)) and one downregulated TF (ETS proto-oncogene 1, transcription factor (ETS1)), known to be associated with follicular development and selection. These TFs were selected as key candidates for subsequent functional studies aimed at elucidating their regulatory roles in granulosa cell differentiation and follicle selection.

To further elucidate the regulatory roles of these key TFs, we performed an integrative analysis by intersecting their predicted target genes with genes that were simultaneously and significantly upregulated or downregulated in both mRNA-seq and ATAC-seq datasets (i.e., RNA&ATAC-UP and RNA&ATAC-DOWN). As shown in Fig. 6C, 651 upregulated (region I) and 572 downregulated (region II) genes were predicted targets of the upregulated TFs (CREM, ESRRB, NR5A1, and GATA6), 244 upregulated (region III) and 236 downregulated (region IV) genes were potential targets of the downregulated TF, ETS1 (Fig. 6D). These consistently regulated genes across both omics layers are likely to be directly modulated by the identified key TFs.

### Functional pathway enrichment and interaction analysis of core transcription factor target genes

To systematically explore the regulatory roles of core TFs, we performed KEGG pathway enrichment analysis on the four functional gene sets (Regions I–IV) defined in Fig. 6C and D. The results revealed that target genes in region Ⅰ were enriched in pathways related to neurotransmitter synthesis, signal transduction, and steroid metabolism, including the phosphatidylinositol signaling system, dopaminergic synapse, and ovarian steroidogenesis (Fig. S4A). Region Ⅱ was enriched in pathways such as the PI3K-Akt, estrogen, and MAPK signaling pathways, which are associated with cytoskeletal regulation, cell adhesion, and hormone signaling (Fig. S4B). Region Ⅲ showed enrichment in oocyte meiosis, GnRH secretion, circadian entrainment, among others (Fig. S4C). Region Ⅳ genes were primarily enriched in axon guidance, Wnt and MAPK signaling pathways, all of which are closely related to cell adhesion, tissue development (Fig. S4D).

**Fig. 7:**
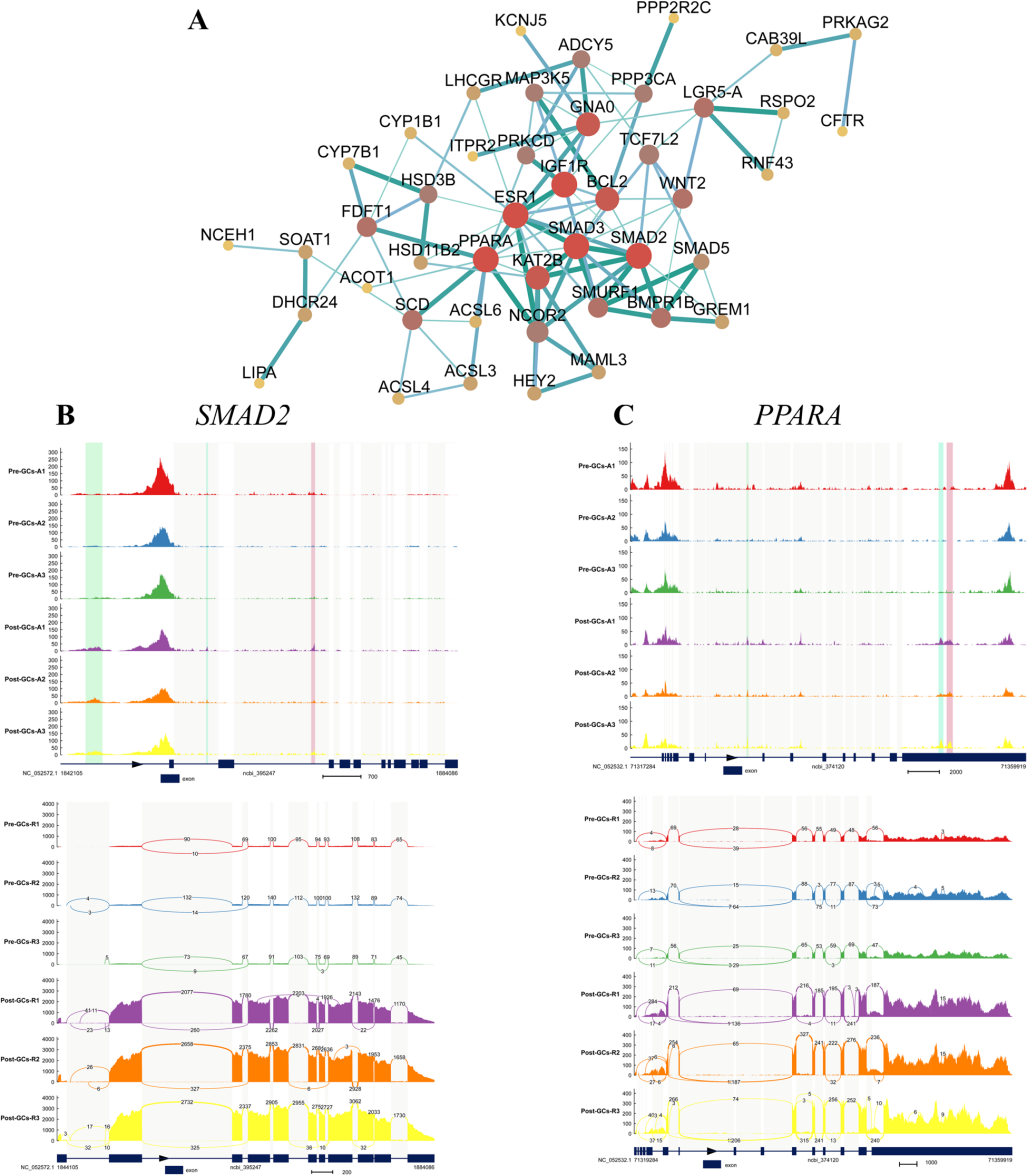
Analysis of upregulated genes in region I. **A** PPI network of target genes enriched in follicle selection-related pathways in within region I. Thicker and darker edges indicate higher interaction confidence, while larger and darker nodes represent proteins with more interactions. **B** and **C** Genome browser track (top) and read coverage (bottom) for *SMAD2* (**B**) and *PPARA* (**C**) showing chromatin accessibility and transcriptional activity at the gene locus

**Fig. 8:**
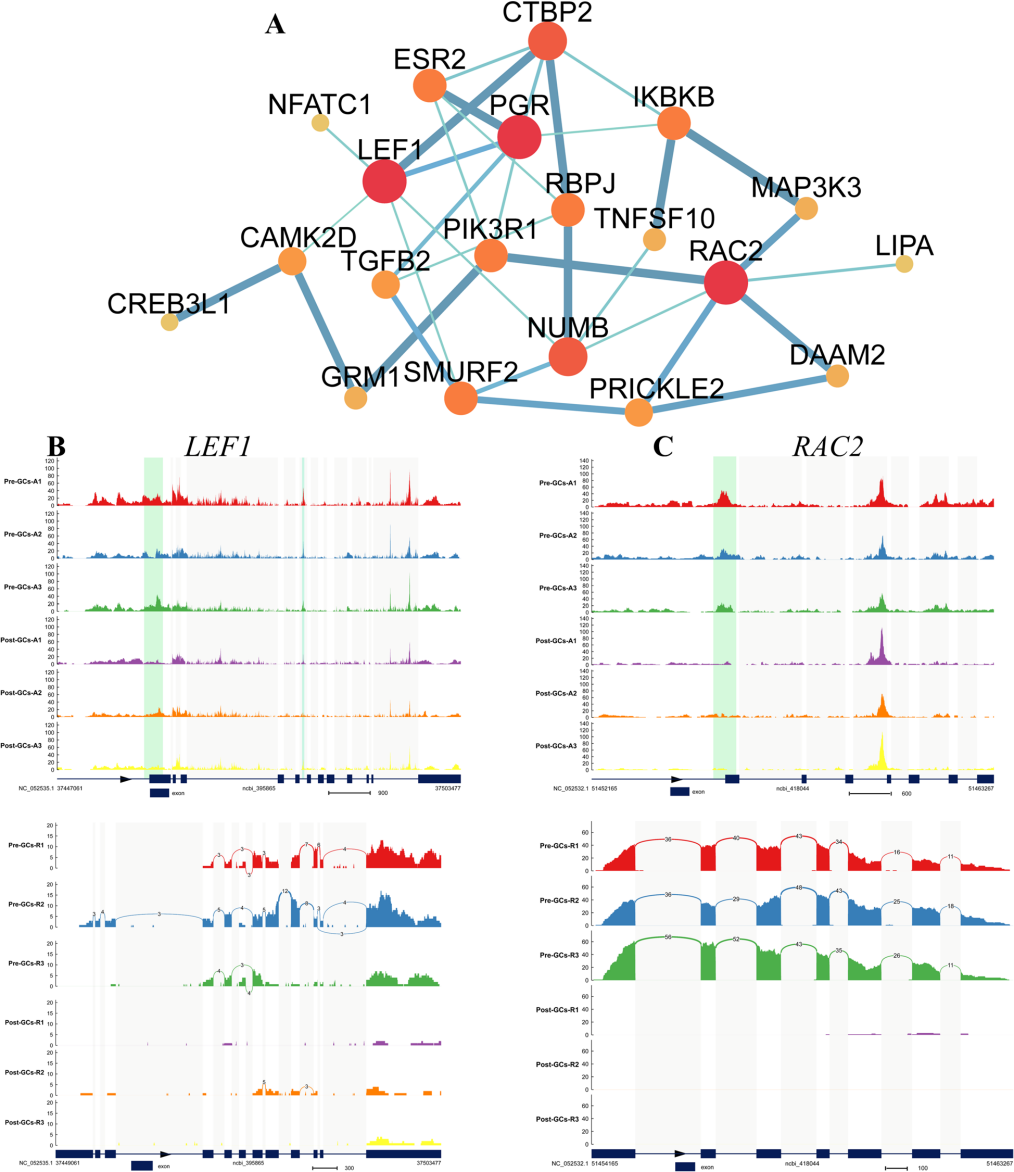
Analysis of downregulated genes in region IV. **A** PPI network of target genes enriched in follicle selection-related pathways in within region IV. Thicker and darker edges indicate higher interaction confidence, while larger and darker nodes represent proteins with more interactions. **B** and **C** Signal enrichment of *LEF1* (**B**) and *RAC2* (**C**) in the differential gene regions from both ATAC-seq (top) and RNA-seq (bottom) data

To further explore the regulatory landscape of follicle selection, we focused on key signaling pathways such as the Wnt, estrogen, and TGF-β pathways (Table S11) within regions Ⅰ and Ⅳ, and constructed PPI networks for the enriched target genes. In region I, upregulated genes such as SMAD family member 2 (*SMAD2*) and peroxisome proliferator-activated receptor alpha (*PPARA*) occupied central positions in the PPI network, suggesting that these genes may be regulated by TFs like NR5A1 and GATA6, and play essential roles in follicle selection (Fig. 7A). The genome browser tracks and read coverage for *SMAD2* (Fig. 7B) and *PPARA* (Fig. 7C) were shown to illustrate their chromatin accessibility and transcriptional activity, providing further insight into their regulatory context. In contrast, region IV featured downregulated genes such as lymphoid enhancer-binding factor 1 (*LEF1*) and ras-related C3 botulinum toxin substrate 2 (*RAC2*), which formed a tightly connected network and may be involved in the negative regulation of follicle selection through the suppressive TF ETS1 (Fig. 8A). Genome browser tracks and read coverage for *LEF1* (Fig. 8B) and *RAC2* (Fig. 8C) were also shown to highlight chromatin accessibility and transcriptional activity in these loci. These findings reveal a dual regulatory mechanism, whereby the target genes of core TFs may exert either activating or inhibitory effects during follicle selection.

### GATA6 promotes cholesterol biosynthesis and progesterone production in Pre-GCs

**Fig. 9:**
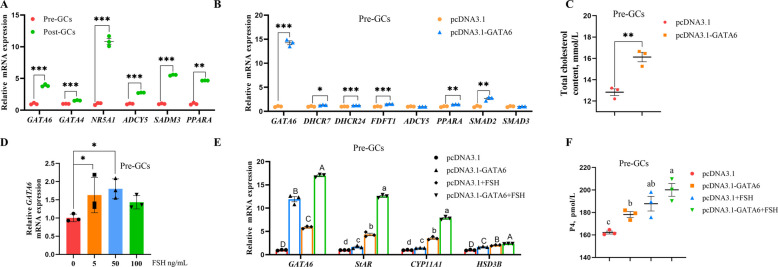
GATA6 regulates cholesterol biosynthesis and progesterone secretion in chicken Pre-GCs. **A** RT-qPCR validation of sequencing results. **B** The effect of *GATA6* overexpression on target gene expression in chicken Pre-GCs. **C** The impact of *GATA6* overexpression on cholesterol synthesis in chicken Pre-GCs. **D** The effect of different concentrations of FSH on *GATA6* mRNA expression in chicken Pre-GCs. **E** and **F** The combined effect of *GATA6* overexpression and FSH treatment on the expression of progesterone-related genes (**E**) and progesterone secretion (**F**) in chicken Pre-GCs

To validate the sequencing results, we randomly selected six genes, including *GATA6*, for RT-qPCR. All six genes were upregulated following follicle selection, consistent in direction with the transcriptomic data (Fig. 9A). Furthermore, several target genes of GATA6 were randomly selected to examine their expression changes. The results showed that GATA6 significantly upregulated the expression of key genes involved in cholesterol biosynthesis, including 7-dehydrocholesterol reductase (*DHCR7*), 24-dehydrocholesterol reductase (*DHCR24*), and farnesyl diphosphate farnesyltransferase 1 (*FDFT1*) (Fig. 9B), thereby promoting cholesterol biosynthesis (Fig. 9C). It also upregulated *PPARA* and *SMAD2*, while having no significant effect on the expression of adenylate cyclase 5 (*ADCY5*) or SMAD family member 3 (*SMAD3*) (Fig. 9B). In addition, FSH increased the expression of *GATA6* mRNA in a dose-dependent manner, with the strongest effect at 50 ng/mL (Fig. 9D). Based on these findings, we combined GATA6 overexpression with 50 ng/mL FSH to treat Pre-GCs. GATA6 overexpression increased the expression of progesterone-related genes, including steroidogenic acute regulatory protein (*StAR*), cytochrome P450 family 11 subfamily A member 1 (*CYP11A1*), and hydroxysteroid 3-beta dehydrogenase (*HSD3B*), and promoted progesterone secretion (Fig. 9E and F). FSH treatment further enhanced the pro-steroidogenic effect of GATA6 (Fig. 9E and F).

## Discussion

To understand the molecular mechanisms underlying follicle selection and granulosa cell differentiation is essential for elucidating ovarian function and reproductive regulation in chickens. Despite substantial progress in transcriptomic studies, the epigenetic landscape and transcriptional regulatory programs that drive these developmental transitions remain incompletely characterized. In this study, for the first time, we employed an integrative multi-omics approach, combining ATAC-seq and mRNA-seq, to comprehensively profile chromatin accessibility and gene expression dynamics in granulosa cells before and after follicle selection. The results identified genes that are specifically expressed before and after follicle selection, stage-specific transcription factor networks and their target genes, and revealed the relationship between chromatin accessibility and transcriptional regulation. Our study reveals the coordination between chromatin accessibility and transcriptional activation or repression during follicle selection, providing valuable insights into the molecular regulation of follicle selection and granulosa cell differentiation, while highlighting key factors that may contribute to these processes.

Motif enrichment analysis of ATAC-seq data uncovered a prominent enrichment of AP-1 family members (e.g., FOS::JUN, FOSL1::JUNB) in upregulated chromatin-accessible regions. Its members can be induced by gonadotrophins and are involved in regulating granulosa cell metabolism, cholesterol biosynthesis, and steroidogenesis, playing pivotal roles in follicle development, cell proliferation and differentiation, angiogenesis, ovulation, and corpus luteum formation [28–30]. Notably, NR5A2 showed the strongest motif enrichment among all identified TFs. As a nuclear receptor, NR5A2 is essential for follicle development and ovulation [31]. It not only activates genes related to steroid biosynthesis, thereby promoting estrogen and progesterone production, but also modulates granulosa cell proliferation, apoptosis, and cytoskeletal remodeling [32–36]. After chicken follicle selection, granulosa cells begin to differentiate and produce large amounts of progesterone [4, 7]. Therefore, the active expression of AP-1 family members and NR5A2 in Post-GCs suggests that they may promote the transcriptional programs associated with steroidogenesis and cell differentiation, playing crucial regulatory roles in the later stages of follicle selection. Conversely, downregulated peaks were significantly enriched for motifs of TEAD1–4, WT1, and other TFs. TEADs, central effectors of the hippo signaling pathway, negatively regulate steroidogenesis in mouse granulosa cells and are repressed by FSH, thereby facilitating steroid production [37, 38]. WT1, known as a repressor of granulosa cell differentiation, inhibits key steroidogenic genes and declines in expression during follicle development [39–42]. These findings support that TEAD1–4 and WT1 may act as suppressive regulators, restraining premature granulosa cell differentiation prior to follicle selection.

mRNA-seq analysis identified 146 genes specifically expressed in Pre-GCs and 40 genes in Post-GCs. These genes may function as key regulators of granulosa cell fate. *CARTPT*, specifically expressed in Post-GCs, is known to inhibit cytochrome P450 family 19 subfamily A member 1 (*CYP19A1*) and reduce estrogen synthesis in cattle [43]. Considering that in chickens, estrogen is synthesized by the theca cells and its levels decline following follicle selection [44, 45], we speculate that the upregulation of *CARTPT* in Post-GCs may inhibit estrogen synthesis in theca cells through a paracrine mechanism, thereby participating in the regulation of follicle selection. This is supported by its negligible expression in theca cells (mean TPM = 0.89) [46]. Similarly, *SSTR5*, induced by FSH and known to repress estrogen and progesterone synthesis through bone morphogenetic protein (BMP) signaling, may participate in the functional transition of granulosa cells [47]. Atonal bHLH transcription factor 1 (*ATOH1*), significantly upregulated in Post-GCs-R, has an undefined role in granulosa cells, but its stage-specific expression pattern suggests its involvement in a previously uncharacterized regulatory pathway. Among the genes highly expressed in Pre-GCs, *IL15* modulates steroidogenesis in human granulosa cells [48], transcription factor 4 (*TCF4*) regulates the cell cycle and proliferation in chicken granulosa cells [49], and *CCR2* is implicated in follicle development in mice [50]. In chicken, these genes may be involved in early granulosa cell activation and proliferation prior to differentiation.

Importantly, several Post-GCs-R-specific genes, including *SSTR5*, *ATOH1*, and solute carrier family 17 member 6 (*SLC17A6*), were not expressed in theca cells, while solute carrier family 5 member 7 (*SLC5A7*) showed only minimal expression [46]. Their granulosa-specific expression and stage-specific upregulation suggest that they may serve as reliable markers of granulosa cell differentiation, offering valuable targets for future functional studies.

Integration of ATAC-seq and mRNA-seq reveals complex relationships between chromatin accessibility and transcription during follicle selection. Although chromatin openness generally correlates positively with gene expression, a subset of genes exhibited discordant patterns, suggesting that expression regulation may not solely depend on chromatin accessibility [51–53], as illustrated by the four-quadrant plot. This phenomenon is also particularly evident in Fig. 5C and D, where a substantial number of DEGs showed no significant changes in chromatin accessibility, implying alternative regulatory mechanisms beyond chromatin openness. These may involve, but are not limited to, non-coding RNA regulation, protein interactions, RNA stability, and other post-transcriptional mechanisms.

To further delineate key regulators, we intersected enriched motifs and DEGs, identifying ESRRB, CREM, NR5A1, GATA6, and ETS1 as core TFs. ESRRB, an orphan nuclear receptor, is highly expressed in pre-ovulatory follicles and rapidly downregulated during ovulation [54]. It may cooperate with estrogen receptor α to modulate target gene expression, playing a pivotal role in ovarian function and potentially serving as a biomarker for ovarian cancer [55, 56]. CREM, a central regulator in the cAMP signaling pathway, is significantly upregulated in chicken granulosa cells prior to ovulation and has been shown to regulate the expression of steroidogenic acute regulatory protein [57], which is consistent with our findings. Based on the transcription factor footprint analysis, CREM (Fig. S5A) and ESRRB (Fig. S5B) exhibit higher chromatin accessibility near their binding sites in the Post-GCs group, which is consistent with their higher mRNA expression levels in pre-ovulatory follicular granulosa cells. This suggests that these two TFs may exert stronger regulatory effects in Post-GCs. In chickens, *NR5A1* is critical for maintaining homeostasis of steroid hormone synthesis by promoting granulosa cell proliferation and inhibiting apoptosis [34]. Its upregulation after chicken follicle selection is consistent with our findings. GATA6 plays essential roles in mouse follicle assembly, granulosa cell differentiation, ovulation, and luteinization [58]. Our results show that GATA6 promotes the expression of key cholesterol biosynthesis genes in chicken Pre-GCs and enhances cholesterol synthesis. This finding is consistent with a previous study [59], showing that GATA6 plays a significant role in lipid regulation and acts as an accelerator of hepatic lipid synthesis in chickens. Furthermore, earlier studies have shown that FSH enhances the expression of *GATA6* transcripts in mice [60], which aligns with our observation that FSH dose-dependently increases the mRNA expression of *GATA6*. Our data also demonstrate that GATA6 regulates the expression of *StAR*, *CYP11A1*, and *HSD3B*, and promotes progesterone production. These findings are consistent with those of Convissar et al. [61], underscoring GATA6's pivotal role in the steroidogenesis pathway. Although ETS1 was identified as a downregulated TF, it is a key regulator of the TGF-β signaling pathway, controlling the expression of TGF-β receptor II [62]. Additionally, ETS1 plays a crucial role in pituitary hormone secretion and contributes to both ovulation and corpus luteum formation [62]. Furthermore, ETS1 enhances the expression of VEGF-A/VEGFR2, promoting the invasiveness of ovarian cancer cells [63]. Identifying these key TFs is crucial for understanding the molecular mechanisms of follicle selection and granulosa cell differentiation in chickens.

Notably, among the target genes of upregulated TFs, 572 were found to be significantly downregulated at both RNA and ATAC levels. Similarly, 244 target genes of downregulated TFs were paradoxically upregulated. This suggests that TFs may function not only as activators but also as repressors. For instance, inducible cAMP early repressor (ICER), an inhibitory isoform of CREM, represses *CYP19A1* expression and estrogen synthesis in rats [64], while RE1-silencing transcription factor (REST) recruits corepressor complexes to silence specific gene programs during development [65].

## Conclusions

In summary, our integrated ATAC-seq and mRNA-seq analyses provide valuable insights into the regulatory landscape underlying follicle selection in chickens, highlighting global chromatin and transcriptional changes. We identify core transcriptional regulators, such as NR5A2, GATA6 and WT1, and delineate their stage-specific target networks that coordinate steroidogenesis and cell-fate transitions. Functional assays further demonstrate that GATA6 promotes cholesterol biosynthesis and progesterone production in Pre-GCs, thereby enhancing steroidogenic capacity during granulosa cell differentiation. Together with a set of stage-specific marker genes for granulosa cell fate, these findings advance our understanding of the epigenetic and transcriptional regulatory mechanisms of follicle selection and provide a basis for future functional and network-level studies in avian reproduction.

## Supplementary Information

Additional file 1: Table S1. Primer used in this study. Table S2. Alignment statistics of ATAC-seq data to the reference genome. Table S3. Table of peak counts for each sample. Table S4. Alignment statistics of mRNA-seq data to the reference genome. Table S5. Base composition statistics table. Table S6. Statistical summary of genetic testing. Table S7. 146 DEGs specific to Pre-GCs-R. Table S8. 40 DEGs specific to Post-GCs. Table S9. Four-Quadrant Plot showing concordant increases in chromatin accessibility and gene expression. Table S10. Four-Quadrant Plot showing concordant decrease in chromatin accessibility and gene expression. Table S11. KEGG pathway enrichment analysis for parts I–IV of the study.

Additional file 2: Fig. S1. Quality control and sample-level analysis of ATAC-seq. Fig. S2 Differential peak analysis based on ATAC-seq between groups. Fig. S3. Sample clustering and differential expression analysis derived from mRNA-seq. Fig. S4. KEGG pathway enrichment analysis based on the integrated ATAC-seq and mRNA-seq data. Fig. S5. Transcription factor footprint analysis of *CREM *and *ESRRB *in Pre-GCs-A and Post-GCs-A.

## Data Availability

The raw mRNA-seq and ATAC-seq datasets generated in this study have been deposited in the NCBI Sequence Read Archive under BioProject accession numbers PRJNA1330579 and PRJNA1322141.

## Abbreviations

AP-1: Activator protein 1
ATOH1: Atonal bHLH transcription factor 1
AME: Analysis of Motif Enrichment
ADCY5: Adenylate cyclase 5
BMP: Bone morphogenetic protein
CREM: Cyclic adenosine monophosphate responsive element modulator
CCR2: C-C motif chemokine receptor 2
CARTPT: Cocaine-and amphetamine-regulated transcript prepropeptide
CYP19A1: Cytochrome P450 family 19 subfamily A member 1
CYP11A1: Cytochrome P450 family 11 subfamily A member 1
DEGs: Differentially expressed genes
DAPs: Differentially accessible peaks
DHCR7: 7-Dehydrocholesterol reductase
DHCR24: 24-Dehydrocholesterol reductase
ELISA: Enzyme-linked immunosorbent assay
ESRRB: Estrogen-related receptor beta
ETS1: ETS proto-oncogene 1, transcription factor
F1–F6: Hierarchical follicle
FSH: Follicle stimulating hormone
FBS: Fetal bovine serum
FDFT1: Farnesyl diphosphate farnesyltransferase 1
FRiP: Fraction of reads in peaks
GO: Gene Ontology
GATA6: GATA binding protein 6
HSD3B: Hydroxysteroid 3-beta dehydrogenase
IKZF1: Ikaros family zinc finger 1
IL15: Interleukin 15
ICER: Inducible cAMP early repressor
KEGG: Kyoto Encyclopedia of Genes and Genomes
LH: Luteinizing hormone
LWFs: Large white follicles
LEF1: Lymphoid enhancer-binding factor 1
NR5A1: Nuclear receptor subfamily 5 group A member 1
NR5A2: Nuclear receptor subfamily 5 group A member 2
Pre-GCs: Granulosa cells of pre-hierarchical follicles
Post-GCs: Granulosa cells of hierarchical follicles
PPI: Protein–protein interaction
PCA: Principal component analysis
PPARA: Peroxisome proliferator-activated receptor alpha
RT-qPCR: Reverse transcription real-time quantitative PCR
REST: RE1-silencing transcription factor
RAC2: Ras-related C3 botulinum toxin substrate 2
SEM: Standard error of the mean
SSTR5: Somatostatin receptor 5
SMAD2: SMAD family member 2
SMAD3: SMAD family member 3
StAR: Steroidogenic acute regulatory protein
SWFs: Small white follicles
SYFs: Small yellow follicles
SLC17A6: Solute carrier family 17 member 6
SLC5A7: Solute carrier family 5 member 7
TSS: Transcription start sites
TCF4: Transcription factor 4
TFs: Transcription factors
TEAD1–4: Transcriptional enhancer factor domain family members 1–4
WT1: Wilms tumor 1
