# Supplementary material for: Integrated ATAC-seq and mRNA-seq analyses on granulosa cells identify key regulators of follicle selection in chickens
Source: J Anim Sci Biotechnol. 2026 Apr 17;17:70. doi: 10.1186/s40104-026-01386-y (PMC13088730; doi:10.1186/s40104-026-01386-y)
Supplement: Supplementary file 2 — Additional file 2: Fig. S1. Quality control and sample-level analysis of ATAC-seq. Fig. S2 Differential peak analysis based on ATAC-seq between groups. Fig. S3. Sample clustering and differential expression analysis derived from mRNA-seq. Fig. S4. KEGG pathway enrichment analysis based on the integrated ATAC-seq and mRNA-seq data. Fig. S5. Transcription factor footprint analysis of CREM and ESRRB in Pre-GCs-A and Post-GCs-A. [file 40104_2026_1386_MOESM2_ESM.docx]

**Supplementary Figures**


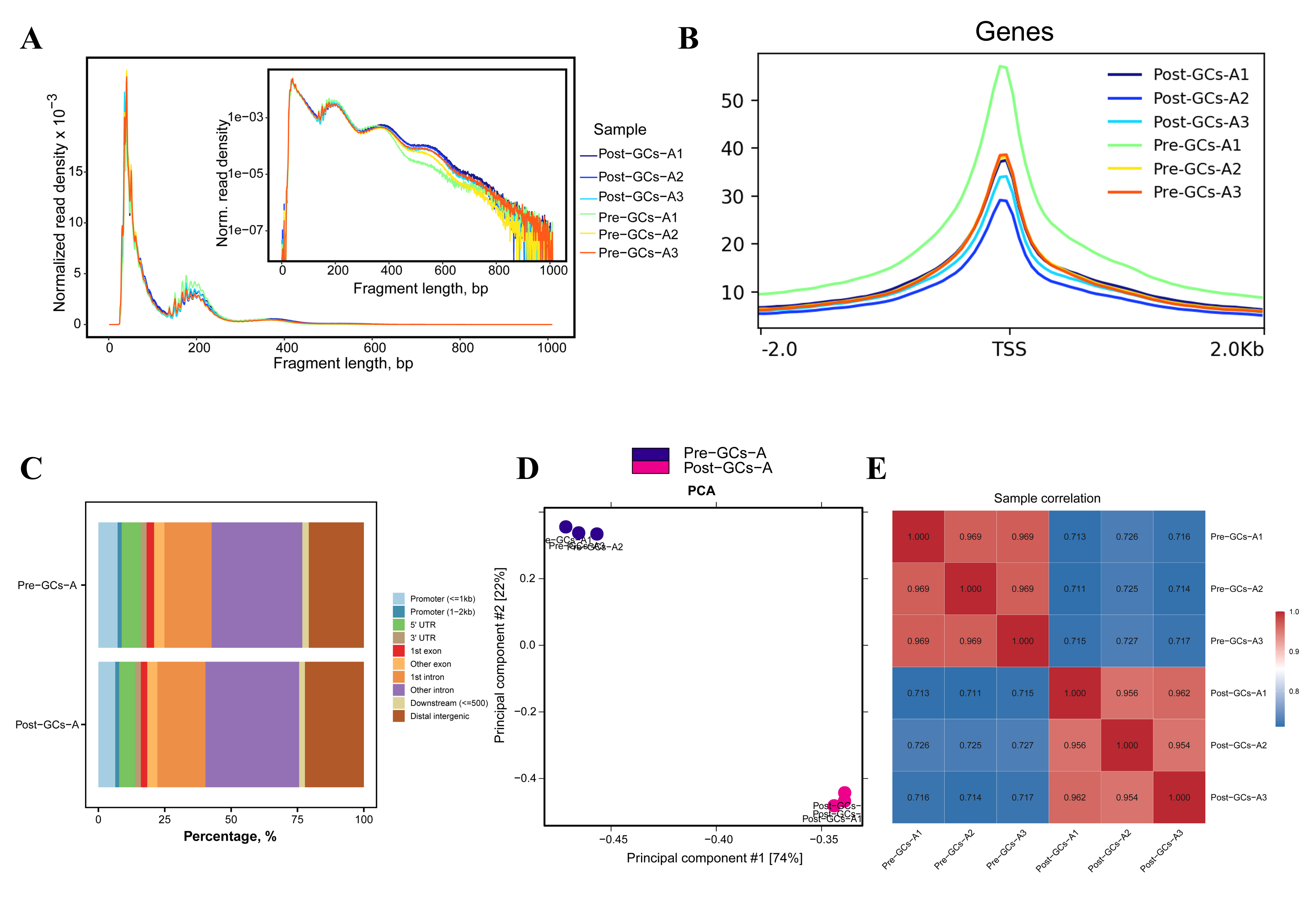


Supplementary Fig. S1 Quality control and sample-level analysis of ATAC-seq. (A) Insert fragment size distribution. (B) Distribution of reads relative to transcription start sites (TSS). (C) Proportional distribution of peaks across genomic functional elements. (D) Principal component analysis (PCA) of samples. (E) Pearson correlation heatmap among samples.


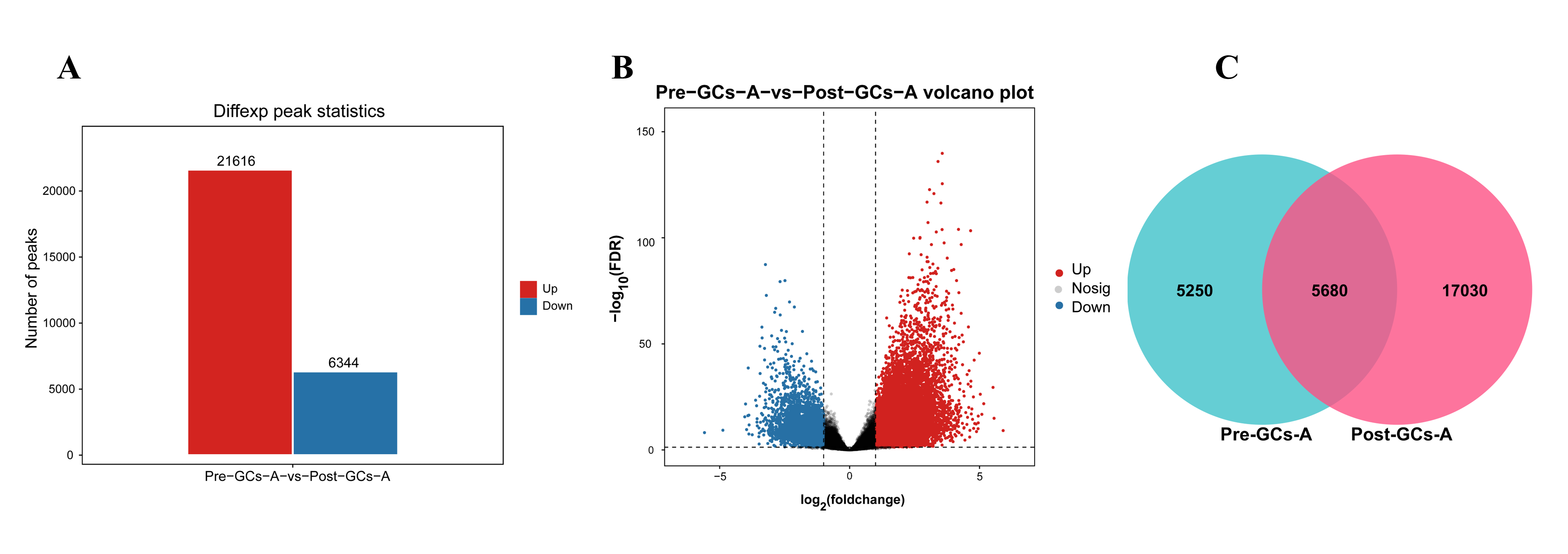


Supplementary Fig. S2 Differential peak analysis based on ATAC-seq between groups. (A) Bar chart of differential peaks between Pre-GCs-A and Post-GCs-A. (B) Volcano plot of differential comparison between Pre-GCs-A and Post-GCs-A. (C) Venn diagram of differential peaks between Pre-GCs-A and Post-GCs-A.


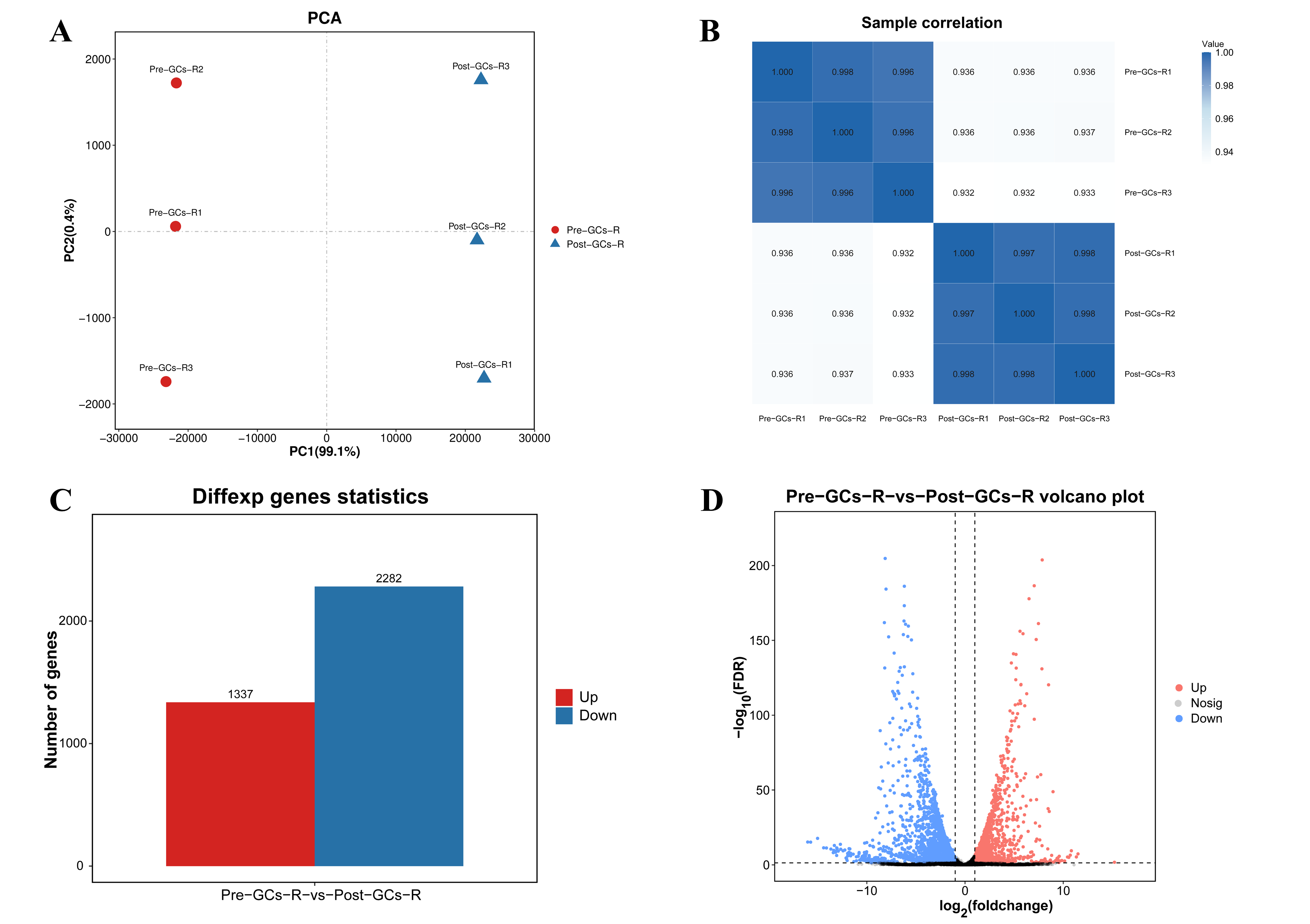


Supplementary Fig. S3 Sample clustering and differential expression analysis derived from mRNA-seq. (A) PCA analysis of all samples. (B) Pearson correlation heatmap of sample replicates. (C) Bar plot of the number of differentially expressed genes between Pre-GCs-R and Post-GCs-R groups. (D) Volcano plot showing differential expression between Pre-GCs-R and Post-GCs-R groups.


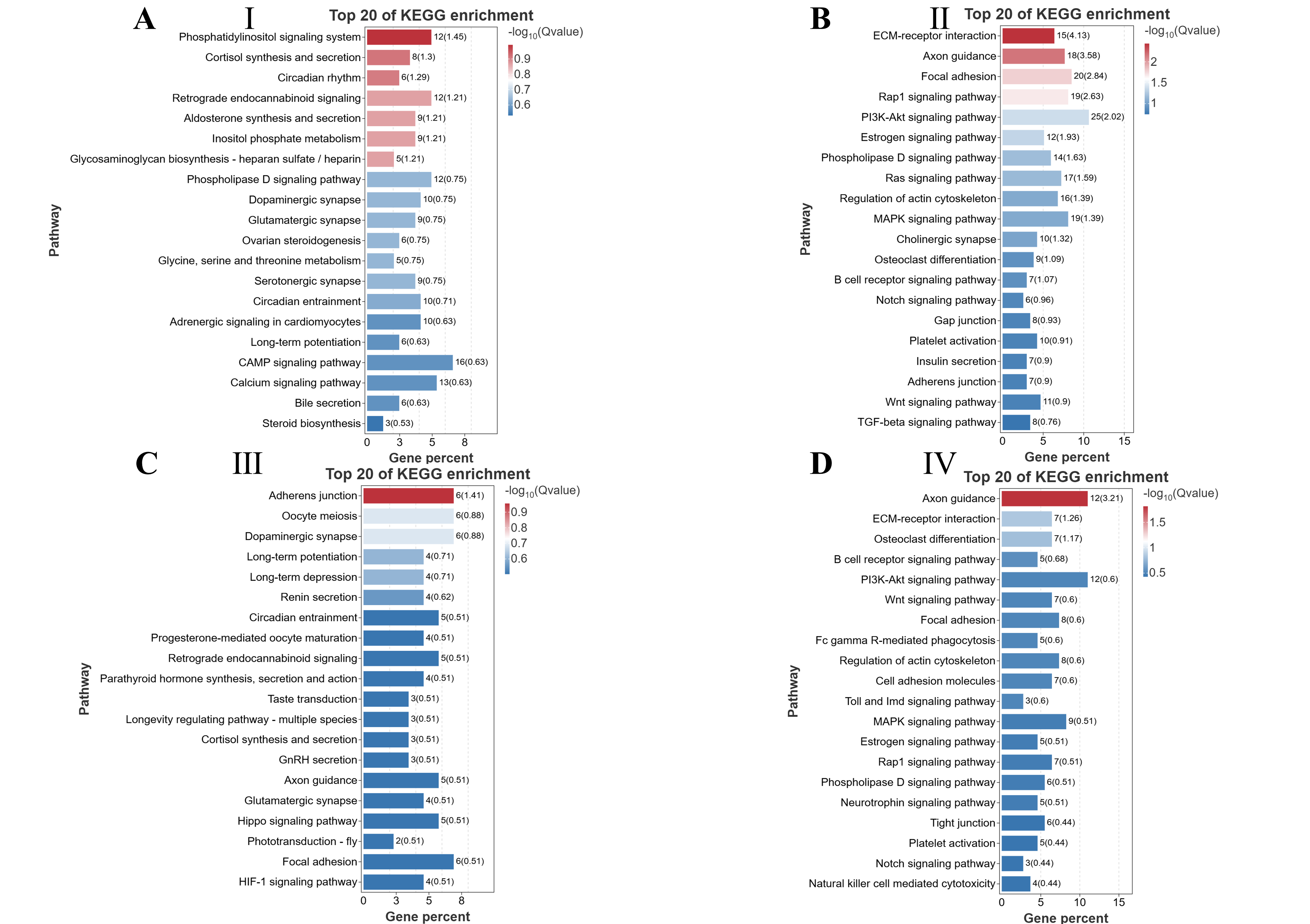


Supplementary Fig. S4 KEGG pathway enrichment analysis based on the integrated ATAC-seq and mRNA-seq data. Panels A–D show the top 20 enriched KEGG pathways for genes in regulatory modules I–IV, respectively, as defined by the integration of chromatin accessibility and gene expression profiles in Fig 6.


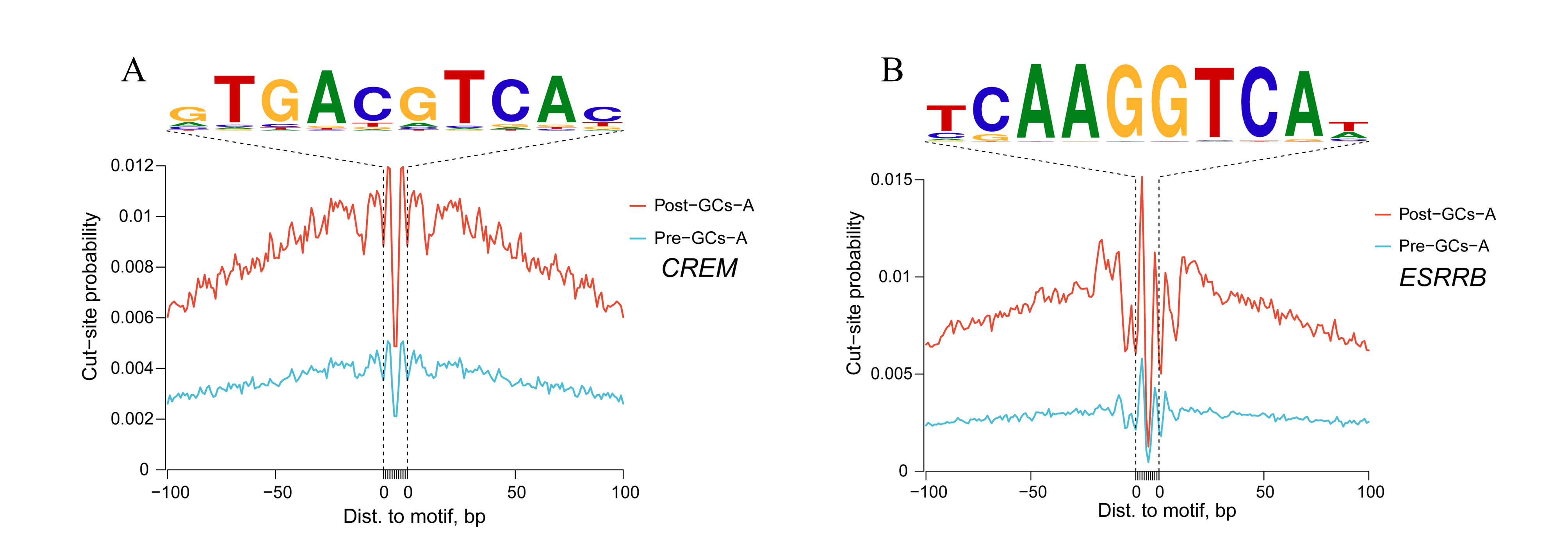


Supplementary Fig. S5 Transcription factor footprint analysis of *CREM* (A) and *ESRRB* (B) in Pre-GCs-A and Post-GCs-A.
